# A genetic variation in fucosyltransferase 8 accelerates HIV-1 disease progression indicating a role for N-glycan fucosylation

**DOI:** 10.1097/QAD.0000000000003689

**Published:** 2023-08-17

**Authors:** Lisa van Pul, Irma Maurer, Brigitte D.M. Boeser-Nunnink, Agnes M. Harskamp, Karel A. van Dort, Neeltje A. Kootstra

**Affiliations:** a Amsterdam Institute for Infection and Immunity; b Department of Experimental Immunology, Amsterdam UMC, location University of Amsterdam, Amsterdam, The Netherlands.

**Keywords:** clinical course, fucosyltransferase 8, HIV-1, pathogenesis, single nucleotide polymorphism

## Abstract

**Objectives:**

Core fucosylation by fucosyltransferase 8 (FUT8) is an important posttranslational modification that impacts components of the immune system. Genetic variations in FUT8 can alter its function and could, therefore, play a role in the antiviral immune response and pathogenesis of HIV-1. This study analysed the effect of a single nucleotide polymorphism (SNP) in FUT8 on the clinical course of HIV-1 infection.

**Design/methods:**

The effect of SNPs in FUT8 on untreated HIV-1 disease outcome were analysed in a cohort of 304 people with HIV-1 (PWH) using survival analysis. Flow-cytometry was used to determine the effect of SNP on T-cell activation, differentiation and exhaustion/senescence. T-cell function was determined by proliferation assay and by measuring intracellular cytokine production. The effect of the SNP on HIV-1 replication was determined by in-vitro HIV-1 infections. Sensitivity of HIV-1 produced in PBMC with or without the SNP to broadly neutralizing antibodies was determined using a TZM-bl based neutralization assay.

**Results:**

Presence of the minor allele of SNP rs4131564 was associated with accelerated disease progression. The SNP had no effect on T-cell activation and T-cell differentiation in PWH. Additionally, no differences in T-cell functionality as determined by proliferation and cytokine production was observed. HIV-1 replication and neutralization sensitivity was also unaffected by the SNP in FUT8.

**Conclusion:**

SNP rs4131564 in FUT8 showed a major impact on HIV-1 disease course underscoring a role for N-glycan fucosylation even though no clear effect on the immune system or HIV-1 could be determined *in vitro*.

## Introduction

Glycosylation of proteins is a common posttranslational modification that regulates protein conformation, structural integrity and function [1–4]. An important type of glycosylation is fucosylation, in which a fucose group is added to O-glycans, N-glycans or glycolipids [5]. The fucosyltransferase 8 (FUT8) enzyme is uniquely responsible for the addition of a fucose residue to N-glycan core structures, a process also known as core fucosylation [5,6]. The gene for FUT8 is located on chromosome 14q23.3 and is expressed in several tissues including brain, gastrointestinal tract and lymphoid tissues ([7] proteinatlas.org). FUT8 plays a crucial physiological role, as shown by the observation that the absence of *Fut8* in knock-out mice leads to severe growth retardation, respiratory defects and, in majority of the cases, early postnatal death [8]. In humans, a rare inherited metabolic disorder called FUT8-CDG has been described, individuals affected have symptoms that are in part similar to those found in *Fut8* knock-out mice and are caused by mutations in FUT8 [9,10]. N-glycan analysis in serum of affected individuals shows significant deficiencies in total core fucosylated N-glycans.

Core fucosylation of N-glycans has a significant role in the immune response. In human adults, approximately 94% of the glycans of plasma immunoglobulin G (IgG) are core fucosylated [11]. Afucosylation of IgG has been shown to enhance antibody binding to Fc receptors [12,13], which can increase the effectivity of antibody-dependent cellular toxicity (ADCC) [13,14]. A concept which is currently utilized in therapeutic antibodies in anticancer strategies [15]. Although most of the IgGs generated by immune responses are fucosylated, responses against some enveloped viruses such as SARS-CoV-2, dengue and HIV-1 generate predominantly afucosylated IgG, which can have differential impacts on disease outcome [16–20].

Furthermore, changes in fucosylation of the heavily core fucosylated T-cell receptor (TCR) have been shown to influence T-cell development, TCR signalling and activation, and T-cell function [21–24]. Fut8 deficiency in mice led to abnormal T-cell development in the thymus [21], and the loss-of-core fucosylation in this model led to reduced CD4^+^ T-cell activation and inflammatory cytokine production [22,23]. Fut8 was also found to be a regulator of the expression of exhaustion marker PD-1, and inhibition of Fut8 leads to reduced expression of cell-surface PD-1 levels and enhanced T-cell function [24].

Alterations in the immune response due to, for instance, low-level core fucosylation, may impact disease outcome in HIV-1 infection [19,25–29]. In a GWA study previously conducted in our lab, a single nucleotide polymorphism (SNP) in FUT8 that had an impact on the clinical course of HIV-1 infection was identified [30]. In this study, the effect of this SNP in FUT8 on immune response and outcome of untreated HIV-1 infection is investigated.

## Materials and methods

### Study population

The population studied consisted of 365 MSM with HIV-1 participating in the Amsterdam cohort studies (ACS) on HIV-1 and AIDS [31] and has been described previously [30]. Genome-wide genotype data was available for 335 participants and after stratification for confounding effects (Eigenstrat, implemented in Eigensoft) [30], 304 participants remained in our study population. Cryopreserved peripheral blood mononuclear cells (PBMC) obtained at time points 1 and 5 years after seroconversion were available for 60 of the participants for flow cytometry analysis of cell surface marker expression. For 37 out of the 60 participants, a preseroconversion sample was available.

Healthy blood donor samples were obtained through the Dutch national blood bank in Amsterdam, The Netherlands (Sanquin).

The ACS has been conducted in accordance with the ethical principles set out in the declaration of Helsinki. Written informed consent was provided by all participants. The study was approved by the institutional medical ethical committee of the Academic Medical Center and the ethics advisory body of the Sanquin Blood Supply Foundation in Amsterdam.

### Single nucleotide polymorphism characterization

Ninety-two SNPs in the FUT8 (chr. 14) region (Supplementary Table 1) were previously determined in a GWA study using the Illuminia Infinium HumanHap300 BeadChip (Illumina, San Diego, California, USA) [30]. Genotyping of additional samples was performed using PCR and fragment analysis of restriction enzyme digestion. Briefly, using primers FUT8-Fw (5′-GGGTATCTGTGAAGCTAAGCCT-3′) and FUT8-Rev2 (5′- GTGCCTGGATTACTGCTTGAATTTCTAA-3′) DNA was amplified by PCR using the following cycling conditions: 5 min at 94 °C; 30 cycles of 5 s at 94 °C, 10 s at 50 °C and 30 s at 72 °C; 5 min 72 °C. PCR products were then digested with MseI (New England Biolabs, Ipswich, Massachusetts, USA) for 1 h at 37 °C degrees followed by inactivation for 20 min at 65 °C. Digestion product was run on agarose gel to confirm presence of the minor variant of rs4131564. Additionally genotyping for rs4131564 was confirmed by sequencing: PCR amplification was performed using GoTaq Flexi DNA Polymerase (Promega, Madison, Wisconsin, USA) and primer pair FUT8-Fw (5′-GGGTATCTGTGAAGCTAAGCCT-3′) and FUT8-Rev1 (5′- CTGTCCTACAAAGTAGATTGGAGTGC-3′) using the same cycling conditions described above. PCR products were treated with ExoSAP-IT and sequenced using the BigDye Terminator V1.1 Cycle Sequencing kit (Applied Biosystems, Foster City, California, USA) on the ABI 3730XL DNA analyser. Genotyping for rs12342831 (B4GALT1), rs11710456 (ST6GALT1), rs6421315 (IKZF1), rs11847263 (FUT8) and rs2072209 (LAMB1) was performed using ABI TaqMan custom SNP assays (Applied Biosystems).

### Single genome amplification of HIV

Proviral HIV load was determined by single genome amplification (SGA). The following primers were used in a primary PCR HIV Pol-F (5′-TTAGTCAGTGCTGGAATCAGG-3′) and Pol-D (5′-GCTACATGAACTGCTACCAGG-3′), followed by nested PCR using primers Pol-B (5′-TAACCTGCCACCTGTAGTAGCAAAAGAAAT-3′) and Pol-E (5′-ATGTGTACAATCTAGTTGCCA-3′). For each sample, at least 10 PCR reactions were performed using 0.5–50 ng DNA input per reaction. The following cycling conditions were used: 5 min at 94 °C; 35 cycles of 15 s at 94 °C, 30 s at 50 °C and 45 s at 72 °C; 5 min 72 °C. PCR products were visualized on 1% agarose gel. The proviral DNA copy number was calculated a maximum likelihood estimation, assuming that when more than 50% of PCR reactions was negative, each positive reaction contained 1 copy proviral DNA.

### Serum biomarkers

The concentrations of soluble CD163 (sCD163) and intestinal fatty acid-binding protein (iFABP) were measured in plasma of donors using a Duoset ELISA kit (R&D systems, Minneapolis, Minnesota, USA).

### Serum N-glycan profiling

Fucosylation of N-glycans was determined in serum of blood donors using DNA sequencing equipment-Fluorophore Assisted Carbohydrate Electrophoresis (DSA-FACE) technology.

### Flow cytometry analyses

The expression of cellular markers on CD4^+^ and CD8^+^ T cells were determined by flow cytometry using cryopreserved PBMC. T-cell activation levels were determined by assessing CD38^+^ and HLA-DR^+^ co-expression or Ki-DR^+^ expression; T-cell exhaustion was determined by PD-1^+^ expression; T-cell senescence was determined by CD57^+^ expression or the loss of CD27/CD28 expression (CD27^–^/CD28^–^). For surface marker detection, PBMC were stained with fluorescent-labelled antibodies for 30 min at 4 °C in the dark followed by a wash step with PBS supplemented with 0.5% BSA. For intracellular staining, PBMC were fixed (FACS Lysing Solution; BD Biosciences, Franklin Lakes, New Jersey, USA), permeabilized (FACS Permeabilization Buffer; BD Biosciences) and stained intracellularly using fluorescently labelled antibodies for 30 min at 4 °C in the dark followed by a wash step with PBS supplemented with 0.5% BSA. Before flow cytometry analysis, PBMC were fixed with CellFIX (BD Biosciences). The following fluorescent-labelled antibodies were used: CD4 APC, CD8 PerCP, CD38 PE, CCR7 PE, HLA-DR FITC, CD57 APC, CD28 PerCP-Cy5.5, CD27 PerCP-Cy5.5, CD45RA PE-Cy7, CD8 PB, CD4 PE-Cy7, CD3 V500 (BD Biosciences), CD4 APC-eFluor780, CD27 APC-eFluor780, PD-1 PE (eBioscience, San Diego, California, USA) and Ki67-FITC (Immunotech, Marseille, France). Fluorescence was measured on the FACS Canto II (BD Biosciences) and marker expression levels were analysed using FlowJo Version 10.8.1 (TreeStar, Ashland, Orlando, USA).

### Proliferation assay

Proliferation assays were performed using cryopreserved PBMC. Cells were stained with CellTrace Violet (ThermoFisher, Waltham, Massachusetts, USA) according to the manufacturer's instruction and cultured for 6 days in the presence of medium alone Roswell Park Memorial Institute (RPMI) 1640 supplemented with 10% heat-inactivated human pool serum (HPS), penicillin (100 U/ml), streptomycin (100 U/ml)), anti-CD3 and anti-CD28 (2 μg/ml), HIV-1 gag peptide pool (2 μg/ml; NIH AIDS reagent program, 12425) or HCMV pp65 peptide pool (2 μg/ml; NIH AIDS reagent program, 11549). After 6 days, cells were stained for cell surface markers and analysed by flow cytometry as described above. The following fluorescently labelled antibodies were used CD3 FITC, CD4 PerCP-Cy5.5 (BD Bioscience) and CD8 APC (BioLegend, San Diego, California, USA). Proliferation in the CD4^+^ and CD8^+^ T-cell population was determined as the proportion of cells that were able to proliferate in response to stimulation. Fluorescence was measured on the FACS Canto II (BD Biosciences) and marker expression levels were analysed using FlowJo Version 10.8.1 (TreeStar).

### Intracellular cytokine production

To determine intracellular cytokine production, PBMC of PWH were cultured in RPMI 1640 supplemented with 10% FCS, penicillin (100 U/ml), streptomycin (100 U/ml), anti-CD28 (2 μg/ml) and anti-CD29 (1 μg/ml) alone or stimulated with either HIV-1 clade B gag peptide pool (2 μg/ml; NIH AIDS reagent program, 12425) or with staphylococcal enterotoxin B (SEB; 2 μg/ml). To all cultures, FitC-labelled CD107a, BD GolgiStop and BD GolgiPlug (BD Biosciences) were added simultaneously. After 6 h incubation at 37 °C, cells were washed with PBS supplemented with 0.5% BSA and then stained extracellularly for 30 min at 4 °C in the dark with CD3 V500, CD4 BV650 and CD8 PB (BD Biosciences) followed by another wash step. For intracellular staining, cells were fixed and permeabilized using the BD cytofix/cytoperm kit (BD Biosciences) followed by staining for 30 min at 4 °C in the dark using the following fluorescently labelled antibodies: IL-2 PE, IFN-y APC AlexaFluor750, Mip1B PE-Cy7 and TNF-a AlexaFluor700. Before flow cytometry analysis, PBMC were washed using PBS supplemented with 0.5% BSA and fixed with CellFIX (BD Biosciences). Fluorescence was measured on the LSRFortessa (BD Biosciences) and marker expression levels were analysed using FlowJo Version 10.8.1 (TreeStar).

### Production of HIV-1 in peripheral blood mononuclear cells

Donor PBMC were stimulated for 3 days with Phytohemagglutinin (PHA; 1 μg/ml) in Iscove's modified Dulbecco medium (IMDM) supplemented with 10% FCS, penicillin (100 U/ml), streptomycin (100 U/ml) and ciproxin (5 ug/ml) at 4 × 10^6^/ml cells. Stimulated PBMC were then plated at 3 × 10^6^ cells per well in IMDM supplemented with IL-2, 10% FCS, penicillin, streptomycin and ciproxin and infected with HIV-1 NL4.3-BaL. At day 7, cell pellets and viruses were harvested. Tissue culture infectious doses (TCID_50_) of the produced viruses were determined on TZM-bl (4000 cells/well). Luminescence was determined after 3 days using an in-house luciferase assay: 25 μl of luciferase substrate (0.83 mmol/l ATP, 0.83 mmol/l dluciferin (Duchefa Biochemie B.V., Haarlem, The Netherlands), 18.7 mmol/l MgCl_2_, 0.78 μmol/l Na_2_H_2_P2O_7_, 38.9 mmol/l Tris (pH 7.8), 0.39% (v/v) glycerol, 0.03% (v/v) Triton X-100, and 2.6 μmol/l dithiothreitol) was added per well. Luminescence was measured using a luminometer (Berthold Technologies, Bad Wildbad, Germany) and is expressed as Relative light units (RLU).

### In-vitro HIV-1 replication kinetics in peripheral blood mononuclear cells

PBMC were isolated from buffy coats from 32 blood donors. PBMC at a concentration of 5 × 10^6^/ml were cultured with IL-2 (20 U/ml) for 3 days in IMDM supplemented with 10% FCS, penicillin (100 U/ml), streptomycin (100 U/ml) and ciproxin (5 ug/ml) followed by infection with HIV-1 NL4.3 or NL4.3-BaL at an multiplicity of infection (MOI) of 0.005. HIV-1 replication was determined by measuring Gag p24 production in the culture supernatant after 8 days by an in-house p24 ELISA.

### Neutralization assay

Neutralization sensitivity to broadly neutralizing monoclonal antibodies (bnAb) VRC01 and 2G12 was determined using a TZM-bl based assay. For the neutralization assays, an inoculum of 200 TCID_50,_ as determined on TZM-bl cells, was used from each virus produced in donor PBMC. Viruses were incubated for 1 h at 37 °C with two-fold serial dilutions of VRC01 (range 0.04–2.5 ug/ml) and 2G12 (range 0.4–25 ug/ml). Virus–antibody mixtures were added to 5000 TZM-bl cells in triplicate. After 48 h, luminescence was determined by an in-house luciferase assay as described above.

### Statistical analysis

Survival analysis was performed by Kaplan–Meier and Cox proportional-hazard analysis. Differences in CD4^+^ T-cell counts, and plasma viral load were analysed using two-sided independent sample *t* test. Differences in immune marker expression between groups were determined by Mann–Whitney *U* test. Neutralizing antibody titres were calculated by nonlinear regression as the concentration at which 50% of the infectivity was inhibited (IC50). Statistical analyses were performed using IBM SPSS Statistics v.28 (IBM, Armonk, New York, USA) and GraphPad Prism version 9.1.0 (GraphPad Software, San Diego, California, USA).

## Results

**Fig. 1:**
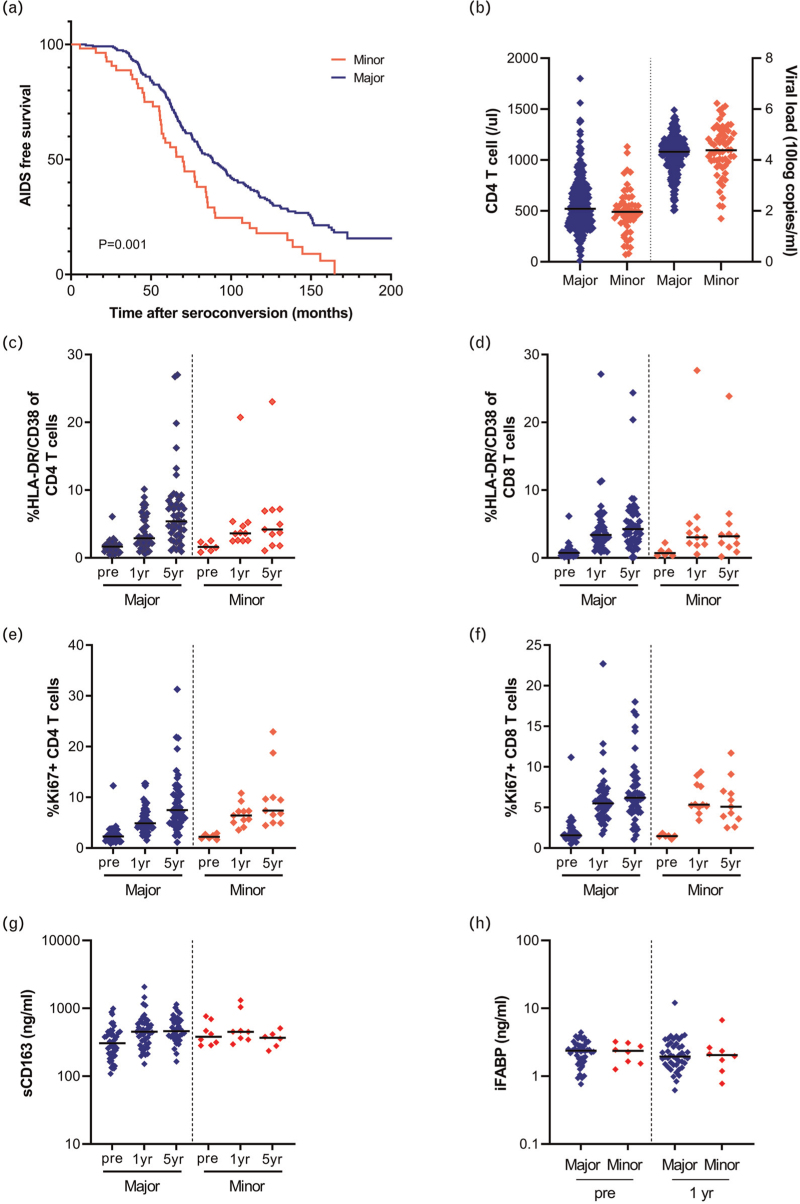
The effect of a single nucleotide polymorphism in FUT8 on HIV-1 disease progression, CD4^+^ T-cell count, viral load at setpoint, T-cell activation and serum sCD163 and iFABP levels.

We analysed 92 SNPs in the *FUT8* gene region for their effect on HIV-1 disease progression in the Amsterdam cohort studies (ACS). SNP rs4131564 was associated with an accelerated disease progression (*P* < 0.001), after correction for multiple testing, using a dominant model with AIDS-defining events according to the 1993 Centres for Disease Control definition (Fig. 1a and Supplementary Table 1). The 50% survival rate of people with HIV-1 (PWH) carrying the minor allele was reached 18.6 months earlier compared with PWH homozygous for the major allele of rs4131564. The accelerated disease progression in carriers of the minor allele of rs4131564 was independent of CCR5Δ32 genotype and HLA-B∗57 genotype as determined by multivariable Cox regression analysis (Table 1). In addition, no association between the presence of rs4131564 and the frequency of HIV nonprogressors was observed using the chi-square test of independence [*χ*^2^(1, *n* = 304) = 0.237, *P* = 0.811]. The minor allele of rs4131564 was not associated with CD4^+^ T-cell counts in blood or viral load in plasma measured at set point (defined as the relatively stable level 18–24 months after seroconversion; Fig. 1b). Moreover, no significant difference in proviral HIV-1 DNA copy number in PBMC was observed between individuals carrying the minor and the major variant of rs4131564 (Supplementary Figure 1).

(a) Kaplan–Meier survival analysis for genetic variation rs4131564 in FUT8 with time in months from seroconversion to progression to AIDS as defined by the CDC in 1993. (b) Viral load and CD4^+^ T-cell counts at set point for major and minor carrying PWH. Proportion of activation (CD38^+^/HLA-DR^+^) in CD4^+^ (c) and CD8^+^ (d) T-cell population in PWH. Proportion of proliferating T cells as determined by expression of Ki67 in CD4^+^ (e) and CD8^+^ (f) T-cell population in PWH. Concentration sCD163 (g) and iFABP (h) in serum of PWH. Time points: before seroconversion (pre), 1 year and 5 years post seroconversion. PWH, people with HIV.

**Table 1:** Multivariate analysis for the progression to AIDS (Center for Disease Control 1993) for the presence of the minor allele of rs4131564, CCR5-Δ32 genotype and HLA-B^∗^57 allele.

AIDS (CDC 1993)				
Genotype	*P* value	Odds ratio	95% CI	
			Lower	Upper
rs4131564	0,014^a^	0,659	0,473	0,919
CCR5Δ32	<0,001^a^	0,482	0,337	0,691
HLA-B^∗^57	0,001^a^	0,329	0,168	0,646

95% CI, 95% confidence interval.

a Significant after Bonferroni correction for multiple testing. CDC, Center for Disease Control.

Heightened T-cell activation is associated with a more rapid HIV-1 disease progression [28]. The association between the SNP in FUT8 and T-cell activation levels, was analysed in a subgroup of 60 ACS participants before HIV-1 infection and at 1 and 5 years after HIV-1 seroconversion. Overall, the T-cell activation as determined by the proportion HLA-DR^+^/CD38^+^ or Ki67^+^ CD4^+^ and CD8^+^ T cell, increased after HIV-1 infection, but no significant differences between PWH carrying the minor allele of rs4131564 and PWH homozygous for the major allele were observed either before or after seroconversion (Fig. 1c–f). Longitudinal serum levels of soluble CD163, a marker for macrophage activation, and the intestinal fatty acid-binding protein (iFABP), a marker for gut mucosal integrity, did not show significant differences in PWH with or without the minor allele of rs4131564 (Fig. 1g and h) or in donors (Supplementary Figure 2).

FUT8 plays an important role in antibody core-fucosylation and genetic polymorphisms in this gene have been associated with IgG fucosylation [32,33]. SNPs previously identified to affect antibody glycosylation, rs12342831 (B4GALT1), rs11710456 (ST6GALT1), rs6421315 (IKZF1), rs11847263 (FUT8) and rs2072209 (LAMB1) [32,33], did not have an effect on HIV-1 disease progression in our study population (Supplementary Table 2). The rs4131564 SNP in FUT8 has not been associated with IgG fucosylation previously and, therefore, it is not expected that the association with accelerated disease progression is linked to antibody fucosylation and functionality. Moreover, N-glycan profiling revealed no differences in fucosylation levels of serum N-glycans in donors between carriers of the minor allele of rs4131564 and carriers homozygous for the major allele (Supplementary Figure 3).

**Fig. 2:**
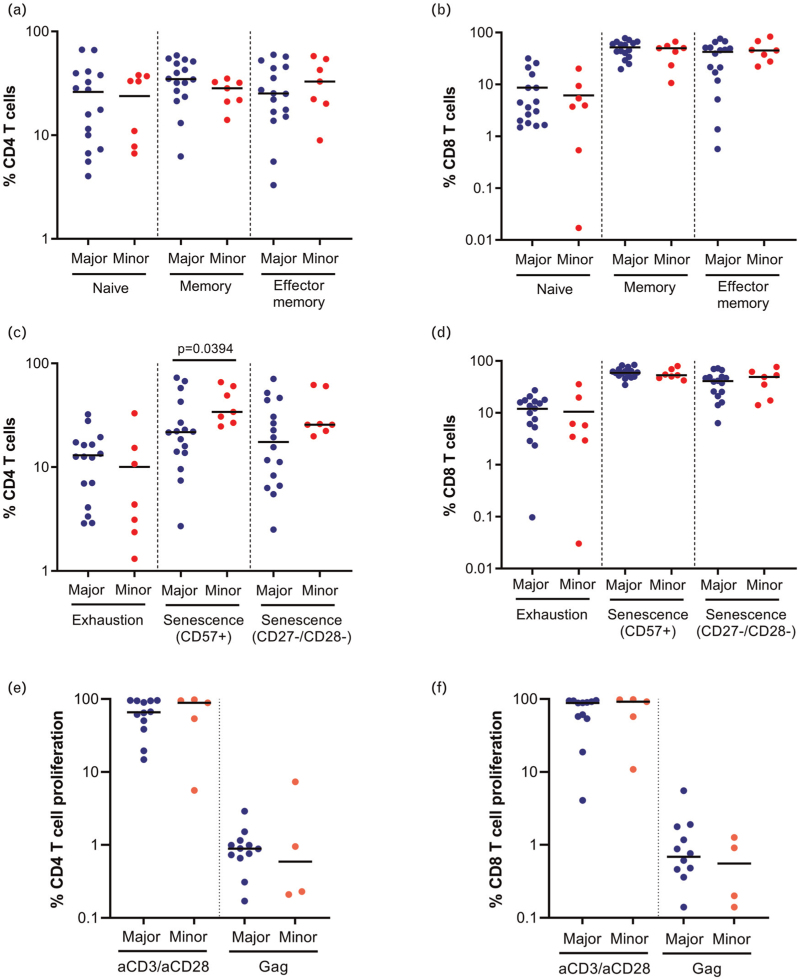
The effect of single nucleotide polymorphism rs4131564 on T-cell differentiation, exhaustion, senescence and proliferation in people with HIV.

CD8^+^ T-cell responses play an important role in the control of HIV-1, and disease progression has been associated with impaired T-cell function and exhaustion [27,34,35]. CD8^+^ T cells recognize HIV-1-infected cells through the interaction between the TCR and HIV-1 peptides presented by MHC class I molecules on the target cell. The TCR is a heavily core-fucosylated glycoprotein and the genetic polymorphism in FUT8 may affect TCR signalling, T-cell differentiation and function [21–23]. Moreover, genes involved in the core fucosylation pathway, including FUT8, have been demonstrated to be involved in the regulation of PD-1 expression and a decrease of FUT8 activity reduced the expression of PD-1 and thus enhance T-cell functionality [24]. To determine the effect of rs4131564 on T-cell differentiation, exhaustion and senescence, PBMC from PWH and blood donors with or without the minor allele of the SNP were analysed by flow cytometry. Donors with the minor allele of rs4131564 had a lower proportion of naive CD4^+^ T cells (Supplementary Figure 4a; *P* = 0.015), whereas no differences in T-cell differentiation were found in PWH (Fig. 2a and b). Exhaustion as determined by PD-1 expression on CD4^+^ and CD8^+^ T cells was not significantly different between the major and minor allele in both PWH and donors (Fig. 2c and d and Supplementary Figure 4c and d). CD8^+^ T-cell senescence as determined by CD57^+^ expression was significantly higher in donors homozygous for the major allele of rs4131564 (Supplementary Figure 4D; *P* = 0.026), and a similar nonsignificant trend was observed for the proportion of CD8^+^ CD27^–^/CD28^–^ T cells. In contrast, CD57^+^ expression was significantly increased on CD4^+^ T cells of PWH carrying the minor allele (Fig. 2c; *P* = 0.039) and a nonsignificant trend towards a higher proportion of CD4^+^ CD27^–^/CD28^–^ T cells was observed in PWH carrying the minor allele.

Flowcytometry analysis of PBMC from PWH carrying the major or minor allele of rs4131564: CD4^+^ and CD8^+^ T-cell differentiation as determined by CD45RA and CD27 expression (a and b); exhaustion as determined by PD-1 expression and senescence as determined by CD57^+^ expression or CD27^–^/CD28^–^ expression (c and d); percentage CD4^+^ and CD8^+^ T-cell proliferation as determined by the loss of CellTrace Violet, upon 6 days of culture with anti-CD3 and anti-CD28 antibody or HIV-1 gag peptide pool stimulation (e and f). PBMC, peripheral blood mononuclear cells; PWH, people with HIV.

**Fig. 3:**
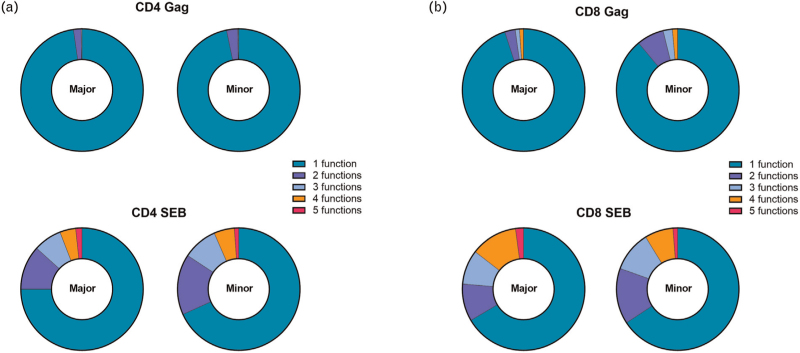
The effect of single nucleotide polymorphism rs4131564 on polyfunctionality of CD4^+^ and CD8^+^ T cells.

To assess whether the functionality of CD4^+^ and CD8^+^ T cells was influenced by the presence of the minor allele of rs4131564, the proliferative capacity of T cells was measured upon anti-CD3/anti-CD28, CMV pp65 peptide pool (CMV-positive donors only) and HIV-1 gag peptide pool stimulation (in PWH only). Results demonstrated comparable levels of proliferation upon stimulation irrespective of the presence of the minor rs4131564 allele in both donors (Supplementary Figure 4e and f) as well as PWH (Fig. 2e and f). In addition, intracellular cytokine production upon stimulation with HIV-1 gag peptide pool or SEB in PWH was determined to assess potential differences in T-cell functionality. No significant differences were found in cytokine production between PWH with the minor allele and those homozygous for the major allele of rs4131564 (Supplementary Figure 5). The polyfunctionality of CD4^+^ and CD8^+^ T cells determined by the ability to produce multiple cytokines simultaneously after stimulation with HIV-1 gag peptide pool or SEB, did not differ between individuals with or without the minor allele of rs4131564 (Fig. 3).

Polyfunctionality of CD4^+^ (a) and CD8^+^ (b) T cells after stimulation with SEB or HIV-1 gag peptide pool in PWH carrying the minor or major allele of rs4131564. PWH, people with HIV; SEB, staphylococcal enterotoxin B.

**Fig. 4:**
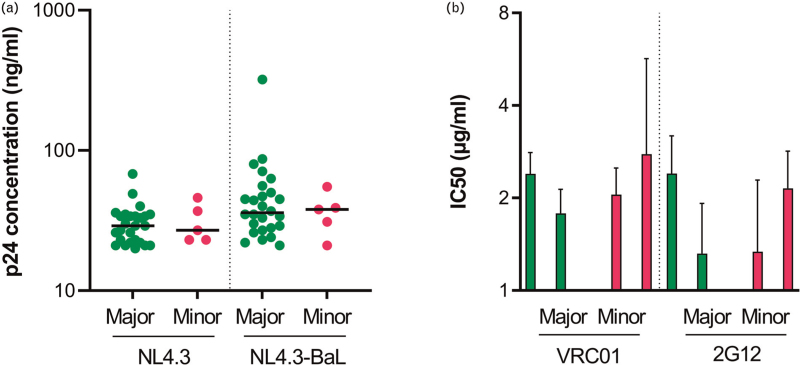
The effect of single nucleotide polymorphism rs4131564 on viral replication and antibody neutralization.

The HIV-1 envelope (Env) is heavily glycosylated, and these glycans are important for protein folding and the interaction with (co-)receptors. Moreover, glycans can shield important conserved regions in Env from the immune system through evasion of neutralization antibodies [36]. The genetic polymorphism in FUT8 may affect Env glycosylation and, therefore, the ability of the virus to replicate in PBMC carrying the minor allele of rs4131564, or their sensitivity to neutralization by antibodies. The effect of SNP rs4131564 in FUT8 on HIV-1 replication was assessed in PBMC from 32 blood donors that were genotyped for the SNP. In-vitro HIV-1 infection of PBMC with NL4.3, a CXCR4-using HIV-1 variant, and NL4.3-BaL, a CCR5-using HIV-1 variant, showed no differences in p24 production indicating that PBMC from donors carrying a minor allele of rs4131564 in FUT8 and donors homozygous for the major allele were equally susceptible to HIV-1 replication (Fig. 4a). Next, we compared the sensitivity of HIV-1 produced in PBMC from donors with (*n* = 2) or without (*n* = 2) the minor rs4131564 allele, to neutralization by VRC01, a bnAb targeting the CD4-binding site, and 2G12, a bnAb targeting the HIV-1 glycan shield. Results showed comparable neutralization, as determined by IC50 values, of HIV-1 produced in PBMC from donors with or without the minor allele of rs4131564 in FUT8 for both antibodies (Fig. 4b).

(a) HIV-1 replication of NL4.3 or NL4.3-BaL, as measured by p24 production, in donor PBMC with or without SNP rs4131564. (B) IC50 (ug/ml) of VRC01 and 2G12 neutralization of NL4.3-BaL produced in PBMC from donors carrying the minor or major allele of rs4131564. Each virus was tested in triplicate and the mean and SD are shown. PBMC, peripheral blood mononuclear cells; SNP, single nucleotide polymorphism.

## Discussion

Core fucosylation is a common posttranslational modification and impacts several elements of the immune response such as antibody and TCR function. Fucosyltransferase 8 is the enzyme that is solely responsible for core fucosylation of N-glycans. Polymorphisms that alter the function or expression levels of FUT8 could, therefore, impact the antiviral immune response and thus the clinical course of HIV-1 infection. Here, we described the effect of a naturally occurring genetic variation in FUT8 on disease outcome of untreated HIV-1 infection. We determined that the minor allele of SNP rs4131564 in FUT8 was associated with accelerated disease progression towards AIDS. The association was found to be independent of the CCR5Δ32 genotype and HLA-B∗57, which have previously been shown to influence disease progression [37,38]. However, the presence of SNP rs4131564 was not associated with CD4^+^ T-cell counts in blood and plasma viral loads at set-point.

Antibody responses play an important role in the immune response against viral infections. The levels of (core-)fucosylation of IgG antibodies have been linked to antibody functionality and disease severity in infection with enveloped viruses such as dengue virus and SARS-CoV-2 [16–18]. Spontaneous control of HIV-1 infection has been associated with higher fractions of afucosylated anti-HIV antibodies [19]. Additionally, untreated controllers and treated progressors had decreased levels of FUT8 expression relative to untreated progressors[19]. Although SNPs in FUT8 have been associated with modulation of IgG core-fucosylation, SNP rs4131564 has not been linked to IgG fucosylation or serum N-glycan fucosylation levels. Moreover, SNPs that have previously been associated with modulation of fucosylation of IgG [rs12342831 (B4GALT1), rs11710456 (ST6GALT1), rs6421315 (IKZF1), rs11847263 (FUT8), and rs2072209 (LAMB1) [32,33]] did not show an association with disease progression in our cohort.

FUT8 has previously been shown to be required for proper T-cell signalling and T-cell activation in mice [21–24]. In HIV-1 infection, T-cell activation levels determined by Ki67 expression or CD38^+^/HLA-DR co-expression as well as PD-1 expression have been associated with untreated disease progression [28,34]. In our cohort of untreated PWH, an increased proportion of T cells expressing Ki67 and CD38/HLA-DR was observed over time. However, no effect of SNP rs4131564 on the levels of T-cell activation before or after HIV-1 seroconversion were observed.

FUT8 has also been shown to influence T-cell differentiation and T-cell activation through altered PD-1 expression [24]. Analysis of the T-cell compartment in PBMC of blood donors did not reveal differences in T-cell differentiation with the exception of the proportion of naive CD4^+^ T cells, which was significantly lower in individuals carrying the minor allele of rs4131564. However, this observation could not be confirmed in PWH. T-cell exhaustion as determined by PD1 expression, did not differ between individuals with or without the minor allele of rs4131564 in donors or PWH. Interestingly, CD8^+^ T-cell senescence (CD57^+^) in donors with the minor allele was found significantly lower whereas CD4^+^ T-cell senescence (CD57^+^) in PWH was significantly increased in individuals carrying the minor allele of rs4131565, which could indicate aberrant TCR signalling. However, analysis of T-cell functionality as determined by proliferation, cytokine production and polyfunctionality upon TCR triggering did not reveal an effect of SNP rs4131564 in either PWH or donors.

As the HIV-1 envelope is heavily glycosylated, we investigated whether virus replication or sensitivity to antibody neutralization was altered by the SNP. Virus replication and neutralization sensitivity of the virus did not appear to be affected by the presence of SNP rs4131564 in FUT8.

In contrast to the in-vivo survival analysis, our in-vitro immunological and virological assays did not reveal clear differences between individuals with or without the minor allele of rs4131564. In most of these assays, stimulation of the cells is required, this may mask the minor effects of the SNP on, for example, TCR signalling, viral envelop fucosylation and viral replication. Indeed TCR stimulation using CMV pp65 peptides, one of the mildest stimulations used here, showed the largest difference in proliferation between the genotypes. Therefore, it cannot be excluded that minor differences in gene function associated with the SNP, that are not measurable in the in-vitro assays, might explain the effect of the SNP on disease progression over the complete course of infection.

A limitation of the study is the relatively small sample size of the cohort. Studies with Fut8-deficient mice show that FUT8 is important in several T-cell processes and functioning [21–23]. However, here we are investigating the potential effect of a SNP, of which the effects could accumulate over time, and not a complete absence of FUT8. Indeed, no disease phenotype or functional impairment has been associated with SNP rs4131564 indicating that the effect of this genetic variation on functionality and expression of FUT8 is minor. In this study, the effect of the SNP in FUT8 on fucosylation levels in serum were investigated and showed no differences. However, the effect of rs4131564 on fucosylation levels in tissues, such as the gut, were not determined. Altered core fucosylation of intestinal epithelial cells in mice has previously been implicated in infection with *Salmonella* Typhi, which was correlated with a change in gut microbiota [39]. In treated HIV-1 infection, low levels of gut fucosylation have been associated with a less diverse gut microbiome, higher levels of gut-associated HIV-DNA levels as well as increased inflammation [40]. Although serum levels of gut integrity marker iFABP did not reveal differences with or without SNP, future investigations on the impact of rs4131564 on HIV-1 disease course should ideally include assessment of core fucosylation in gut tissue samples as well as microbiome analysis. Natural killer (NK) cell and macrophage function through ADCC and cytokine release have been linked to antibody fucosylation levels [41] however, here the function was not studied directly. As there is no association of SNP rs4131564 with antibody fucosylation [32,33] and we did not observe an effect of SNPs previously shown to be associated with fucosylation of IgG on disease progression, we do not expect that the accelerated HIV-1 disease progression associated with SNP rs4131564 is because of impaired NK or macrophage function. Furthermore, no difference in the levels of macrophage activation marker sCD163 in the serum of PWH with or without SNP rs4131564 were found.

In conclusion, the presence of the minor allele of rs4131564 in FUT8 showed an association with accelerated HIV-1 disease progression. We were unable to show an effect of the SNP on the function of the immune system nor on viral replication and neutralization *in vitro*. However, minor effects of the SNP in FUT8 on gut integrity, antibody functionality and subsequent activation of NK cells and macrophages, as well as T-cell functionality cannot be excluded and could have an accumulative combined effect that may have a major impact over time and on the outcome of HIV-1 infection.

## Acknowledgements

We thank all participants in the Amsterdam Cohort Studies for their contribution. The Amsterdam Cohort Studies on HIV infection and AIDS, a collaboration between the Public Health Service Amsterdam, the Amsterdam UMC of the University of Amsterdam, Medical Center Jan van Goyen and the HIV Focus Center of the DC-Clinics, are part of the Netherlands HIV Monitoring Foundation and financially supported by the Center for Infectious Disease Control of the Netherlands National Institute for Public Health and the Environment.

We would like to thank Marit van Gils and Jonne Snitselaar for kindly providing us with the broadly neutralizing antibodies used in the neutralization assay.

Author contributions: L.v.P. designed study and experiments, performed experiments, analysed and interpreted data and wrote the manuscript; I.M. performed experiments and analysed data; B.B.N. performed experiments and analysed data; A.M.H. provided materials and data management; K.v.D. performed experiments and analysed data; N.A.K. designed the study and the experiments, interpreted data, wrote the manuscript, supervised and acquired funding.

### Conflicts of interest

There are no conflicts of interest.

## Supplementary Material

Supplemental Digital Content
