# Supplementary material for: A genetic variation in fucosyltransferase 8 accelerates HIV-1 disease progression indicating a role for N-glycan fucosylation
Source: AIDS. 2023 Aug 17;37(13):1959–69. doi: 10.1097/QAD.0000000000003689 (PMC10552802; doi:10.1097/QAD.0000000000003689)
Supplement: Supplemental Digital Content [file aids-37-1959-s001.docx]

**Supplementary Table 1 : single nucleotide polymorphisms in the gene regions coding for FUT8 and the effect on HIV-1 progression (AIDS (CDC1993))**

|  |  | **Genotype distribution** | | | | **Cox regression^a^** | | |
| --- | --- | --- | --- | --- | --- | --- | --- | --- |
| **SNP** | **n** | **Maj/Maj** | **Maj/Min** | **Min/Min** | **Frequency of minor allele** | **p-value** | **Odds ratio** | **95.0% CI** |
| rs1003401 | 304 | 130 | 144 | 30 | 0.336 | 0.405 | 0.891 | 0.679-1.169 |
| rs10132229 | 304 | 268 | 33 | 3 | 0.064 | 0.337 | 0.809 | 0.524-1.248 |
| rs10148748 | 304 | 253 | 46 | 5 | 0.092 | 0.853 | 1.037 | 0.708-1.518 |
| rs10149555 | 304 | 114 | 137 | 53 | 0.400 | 0.677 | 1.061 | 0.804-1.399 |
| rs10150177 | 303 | 275 | 26 | 2 | 0.050 | 0.790 | 1.070 | 0.651-1.757 |
| rs1036979 | 303 | 258 | 43 | 2 | 0.078 | 0.219 | 0.776 | 0.518-1.163 |
| rs10483776 | 304 | 218 | 78 | 8 | 0.155 | 0.794 | 1.040 | 0.775-1.396 |
| rs10483781 | 304 | 276 | 28 | 0 | 0.046 | 0.309 | 0.773 | 0.471-1.269 |
| rs10483782 | 304 | 267 | 36 | 1 | 0.063 | 0.419 | 1.186 | 0.784-1.795 |
| rs10483785 | 304 | 85 | 172 | 47 | 0.438 | 0.088 | 1.313 | 0.96-1.796 |
| rs10498517 | 304 | 271 | 33 | 0 | 0.054 | 0.876 | 1.035 | 0.671-1.598 |
| rs10498522 | 304 | 256 | 47 | 1 | 0.081 | 0.272 | 1.229 | 0.851-1.775 |
| rs10873193 | 302 | 113 | 148 | 41 | 0.381 | 0.215 | 0.841 | 0.639-1.106 |
| rs11158590 | 304 | 261 | 41 | 2 | 0.074 | 0.698 | 1.079 | 0.734-1.588 |
| rs11158617 | 304 | 261 | 43 | 0 | 0.071 | 0.496 | 0.874 | 0.594-1.287 |
| rs1147437 | 304 | 136 | 123 | 45 | 0.350 | 0.993 | 0.999 | 0.762-1.309 |
| rs1147443 | 304 | 179 | 98 | 27 | 0.250 | 0.639 | 1.067 | 0.813-1.402 |
| rs1147446 | 304 | 204 | 89 | 11 | 0.183 | 0.102 | 0.783 | 0.583-1.050 |
| rs1147450 | 304 | 78 | 159 | 67 | 0.482 | 0.888 | 0.978 | 0.722-1.326 |
| rs1147455 | 304 | 148 | 128 | 28 | 0.303 | 0.836 | 0.972 | 0.743-1.272 |
| rs1147459 | 304 | 257 | 46 | 1 | 0.079 | 0.285 | 0.814 | 0.558-1.187 |
| rs11621121 | 304 | 132 | 138 | 34 | 0.339 | 0.755 | 0.957 | 0.729-1.258 |
| rs12432431 | 304 | 115 | 147 | 42 | 0.380 | 0.463 | 0.903 | 0.686-1.187 |
| rs12433694 | 304 | 96 | 148 | 60 | 0.441 | 0.990 | 0.998 | 0.749-1.330 |
| rs1256501 | 304 | 116 | 150 | 38 | 0.372 | 0.671 | 1.062 | 0.805-1.400 |
| rs1256515 | 304 | 247 | 53 | 4 | 0.100 | 0.929 | 0.983 | 0.681-1.420 |
| rs1256517 | 304 | 243 | 58 | 3 | 0.105 | 0.618 | 1.093 | 0.771-1.548 |
| rs1256526 | 304 | 129 | 143 | 32 | 0.340 | 0.951 | 1.008 | 0.769-1.323 |
| rs1256531 | 302 | 265 | 37 | 0 | 0.061 | 0.494 | 1.152 | 0.768-1.727 |
| rs1256537 | 298 | 270 | 28 | 0 | 0.047 | 0.344 | 0.787 | 0.479-1.293 |
| rs1256543 | 300 | 242 | 58 | 0 | 0.097 | 0.697 | 0.933 | 0.658-1.322 |
| rs1269068 | 304 | 154 | 127 | 23 | 0.285 | 0.668 | 0.943 | 0.720-1.235 |
| rs1273868 | 304 | 238 | 61 | 5 | 0.117 | 0.716 | 1.063 | 0.766-1.473 |
| rs1273870 | 304 | 245 | 57 | 2 | 0.100 | 0.527 | 1.114 | 0.797-1.558 |
| rs12891561 | 302 | 100 | 143 | 59 | 0.432 | 0.350 | 0.872 | 0.654-1.162 |
| rs1663332 | 304 | 133 | 137 | 34 | 0.337 | 0.973 | 0.995 | 0.759-1.305 |
| rs17103248 | 304 | 193 | 93 | 18 | 0.212 | 0.978 | 1.004 | 0.760-1.326 |
| rs1889814 | 304 | 80 | 159 | 65 | 0.475 | 0.800 | 0.962 | 0.712-1.299 |
| rs1953416 | 301 | 78 | 166 | 57 | 0.465 | 0.172 | 1.250 | 0.907-1.721 |
| rs1953417 | 303 | 276 | 25 | 2 | 0.048 | 0.311 | 0.783 | 0.488-1.256 |
| rs1954610 | 304 | 178 | 106 | 20 | 0.240 | 0.749 | 0.956 | 0.728-1.257 |
| rs1959144 | 304 | 136 | 141 | 27 | 0.321 | 0.646 | 1.066 | 0.812-1.399 |
| rs1961948 | 304 | 256 | 47 | 1 | 0.081 | 0.521 | 1.122 | 0.789-1.594 |
| rs2022841 | 304 | 102 | 143 | 59 | 0.429 | 0.054 | 0.759 | 0.574-1.005 |
| rs2268957 | 304 | 276 | 26 | 2 | 0.049 | 0.309 | 0.783 | 0.488-1.255 |
| rs2300871 | 304 | 276 | 26 | 2 | 0.049 | 0.309 | 0.783 | 0.488-1.255 |
| rs2318305 | 304 | 243 | 57 | 4 | 0.107 | 0.931 | 0.985 | 0.698-1.390 |
| rs2411351 | 304 | 84 | 168 | 52 | 0.447 | 0.130 | 1.275 | 0.931-1.748 |
| rs2411813 | 304 | 231 | 70 | 3 | 0.125 | 0.119 | 1.284 | 0.937-1.760 |
| rs2411822 | 303 | 80 | 168 | 55 | 0.459 | 0.119 | 1.289 | 0.936-1.774 |
| rs2411916 | 304 | 164 | 120 | 20 | 0.263 | 0.334 | 1.142 | 0.873-1.494 |
| rs3742597 | 304 | 194 | 99 | 11 | 0.199 | 0.946 | 1.010 | 0.765-1.334 |
| rs3783711 | 304 | 134 | 144 | 26 | 0.322 | 0.566 | 1.083 | 0.825-1.422 |
| rs3935480 | 304 | 241 | 58 | 5 | 0.112 | 0.608 | 1.093 | 0.779-1.532 |
| rs4073416 | 304 | 120 | 139 | 45 | 0.377 | 0.547 | 1.089 | 0.826-1.435 |
| rs4078408 | 304 | 130 | 136 | 38 | 0.349 | 0.782 | 1.039 | 0.792-1.364 |
| rs4131564 | 304 | 245 | 56 | 3 | 0.102 | **0.001** | 1.716 | 1.233-2.388 |
| rs4902379 | 304 | 275 | 29 | 0 | 0.048 | 0.695 | 0.910 | 0.567-1.460 |
| rs4902399 | 304 | 134 | 144 | 26 | 0.322 | 0.566 | 1.083 | 0.825-1.422 |
| rs4902426 | 304 | 106 | 144 | 54 | 0.414 | 0.428 | 0.892 | 0.673-1.183 |
| rs4902438 | 304 | 92 | 152 | 60 | 0.447 | 0.223 | 0.837 | 0.628-1.115 |
| rs6573604 | 304 | 239 | 59 | 6 | 0.117 | 0.468 | 0.883 | 0.632-1.235 |
| rs6573606 | 304 | 251 | 53 | 0 | 0.087 | 0.179 | 0.774 | 0.533-1.124 |
| rs6573628 | 303 | 257 | 46 | 0 | 0.076 | 0.634 | 0.912 | 0.625-1.331 |
| rs6573635 | 304 | 121 | 130 | 53 | 0.388 | 0.895 | 0.982 | 0.745-1.293 |
| rs6573638 | 304 | 213 | 87 | 4 | 0.156 | 0.194 | 0.822 | 0.612-1.105 |
| rs6573648 | 304 | 163 | 106 | 35 | 0.289 | 0.646 | 0.939 | 0.716-1.230 |
| rs7141711 | 304 | 135 | 135 | 34 | 0.334 | 0.747 | 1.046 | 0.797-1.372 |
| rs7143755 | 304 | 254 | 46 | 4 | 0.089 | 0.805 | 0.952 | 0.647-1.402 |
| rs7144971 | 303 | 132 | 143 | 28 | 0.328 | 0.457 | 1.109 | 0.844-1.459 |
| rs7145500 | 304 | 127 | 147 | 30 | 0.340 | 0.539 | 1.090 | 0.828-1.435 |
| rs7145970 | 304 | 191 | 94 | 19 | 0.217 | 0.214 | 0.835 | 0.628-1.110 |
| rs7147624 | 304 | 206 | 87 | 11 | 0.179 | 0.450 | 1.117 | 0.837-1.491 |
| rs7158071 | 304 | 86 | 158 | 60 | 0.457 | 0.440 | 1.127 | 0.832-1.527 |
| rs7159888 | 304 | 134 | 140 | 30 | 0.329 | 0.887 | 1.020 | 0.778-1.337 |
| rs7160924 | 304 | 186 | 101 | 17 | 0.222 | 0.358 | 0.877 | 0.664-1.160 |
| rs7161123 | 304 | 82 | 172 | 50 | 0.447 | 0.149 | 1.263 | 0.920-1.735 |
| rs743085 | 304 | 84 | 170 | 50 | 0.444 | 0.130 | 1.275 | 0.931-1.748 |
| rs8006948 | 304 | 96 | 147 | 61 | 0.442 | 0.520 | 0.909 | 0.679-1.216 |
| rs8007846 | 304 | 110 | 145 | 49 | 0.400 | 0.448 | 1.116 | 0.841-1.480 |
| rs8010134 | 304 | 145 | 131 | 28 | 0.308 | 0.158 | 1.214 | 0.927-1.590 |
| rs8013361 | 293 | 112 | 139 | 42 | 0.381 | 0.727 | 0.951 | 0.719-1.259 |
| rs8013442 | 304 | 133 | 145 | 26 | 0.324 | 0.623 | 1.071 | 0.815-1.407 |
| rs8017202 | 304 | 275 | 27 | 2 | 0.051 | 0.371 | 0.810 | 0.511-1.285 |
| rs8021889 | 304 | 209 | 86 | 9 | 0.171 | 0.412 | 1.129 | 0.845-1.510 |
| rs878815 | 304 | 117 | 134 | 53 | 0.395 | 0.997 | 1 | 0.76-1.314 |
| rs894917 | 304 | 207 | 86 | 11 | 0.178 | 0.86 | 0.974 | 0.724-1.309 |
| rs894921 | 304 | 136 | 124 | 44 | 0.349 | 0.993 | 0.999 | 0.762-1.309 |
| rs899961 | 304 | 173 | 114 | 17 | 0.243 | 0.79 | 0.964 | 0.734-1.265 |
| rs9323465 | 303 | 92 | 143 | 68 | 0.460 | **0.015** | 0.701 | 0.527-0.933 |
| rs975231 | 304 | 133 | 125 | 46 | 0.357 | 0.865 | 1.024 | 0.781-1.342 |
| rs986201 | 304 | 140 | 121 | 43 | 0.340 | 0.759 | 0.959 | 0.732-1.255 |

^a^Dominant model comparing homozygous major to heterozygous or homozygous minor was used for Cox regression analysis

Maj, Major; Min, Minor; 95.0% CI, 95% Confidence interval


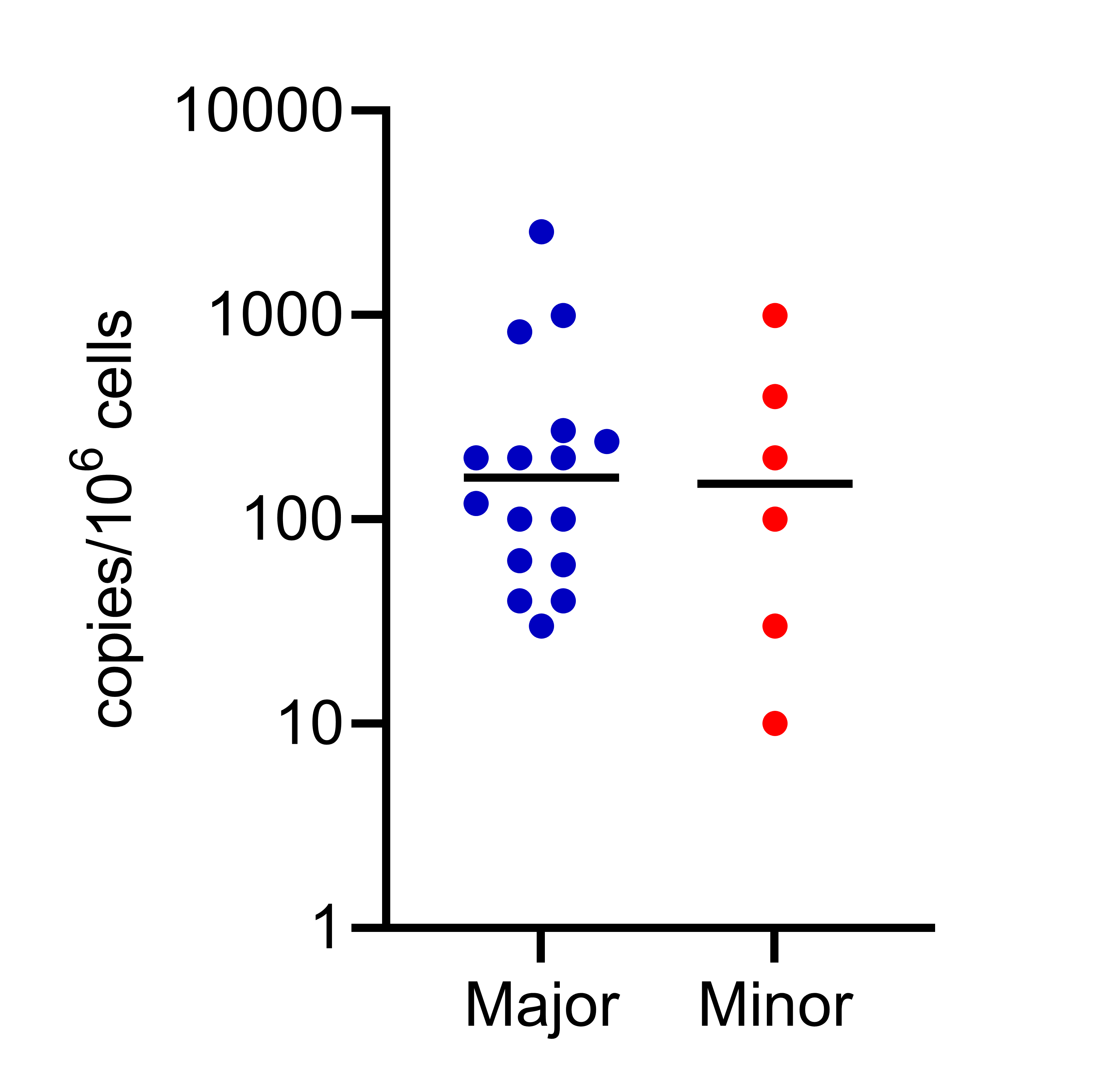


**Supplementary figure 1: HIV-1 proviral DNA load.** Proviral HIV-1 DNA load in copies per million cells, as determined by SGA, in PWH carrying the major or minor allele of rs4131564 in FUT8.

**Supplementary figure 2: The effect of SNP rs4131564 on serum marker expression in blood donors.** Soluble CD163 and iFABP concentrations in serum of blood donors carrying the minor allele of rs4131564 or homozygous for the major allele of rs4131564.

**Supplementary table 2: the effect of SNPs previously identified to affect antibody glycosylation on HIV-1 disease progression.**

| **AIDS (CDC1993)** | | | | | |
| --- | --- | --- | --- | --- | --- |
| **SNP** | **gene** | **n** | **p-value** | **Odds ratio** | **95.0% CI** |
| rs11710456 | ST6GALT1 | 298 | 0.098 | 1.183 | 0.970-1.443 |
| rs6421315 | IKZF1 | 297 | 0.773 | 1.031 | 0.838-1.269 |
| rs12342831 | B4GALT1 | 297 | 0.509 | 1.077 | 0.865-1.341 |
| rs11847263 | FUT8 | 296 | 0.298 | 0.896 | 0.729-1.102 |
| rs2072209 | LAMB1 | 298 | 0.561 | 0.890 | 0.601-1.318 |

95.0% CI, 95% Confidence interval


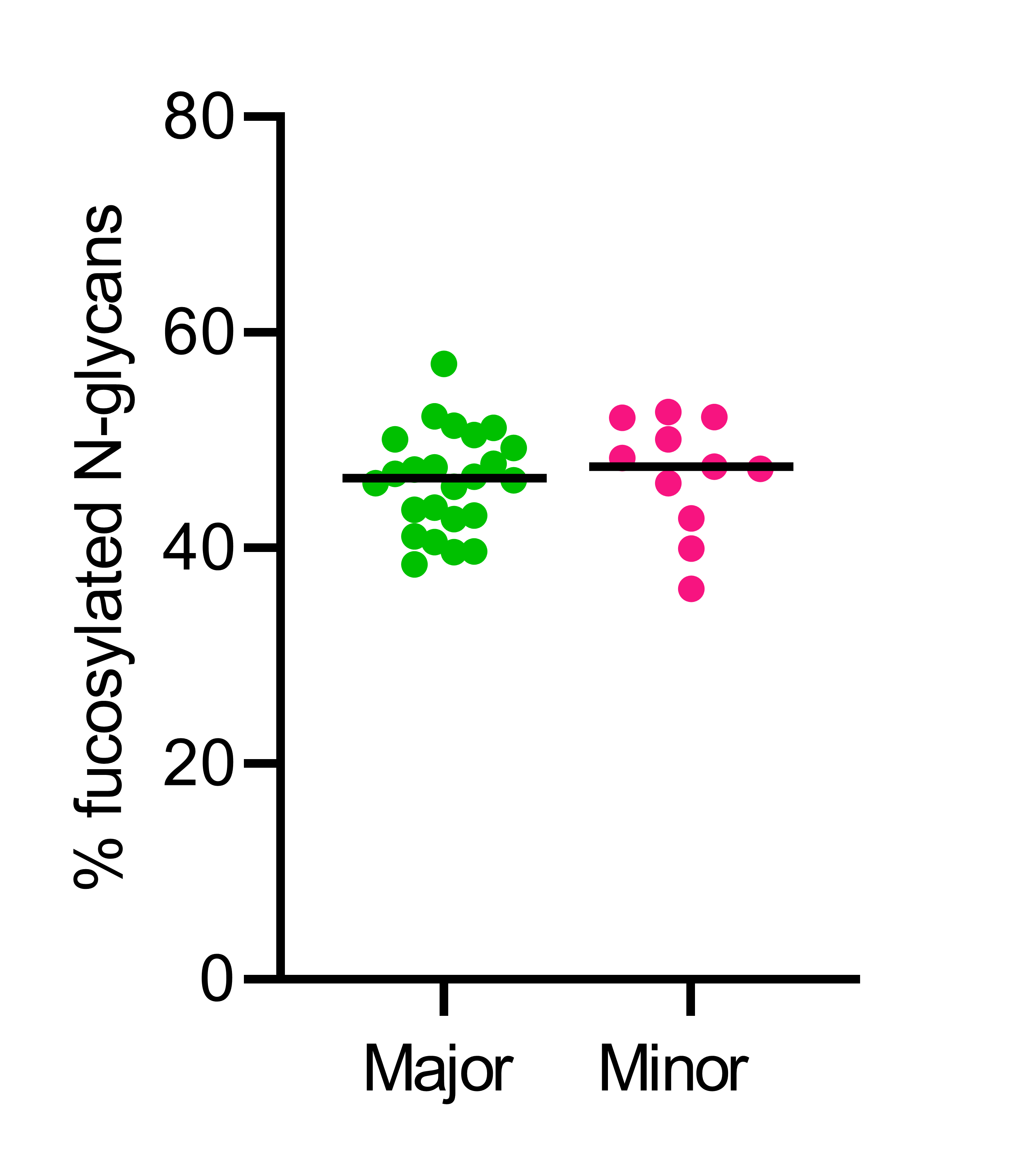


**Supplementary figure 3: N-glycan profiling in serum of blood donors.** Fucosylation levels as determined by DSA-FACE analysis in serum of blood donors carrying the major or minor allele of rs4131564.


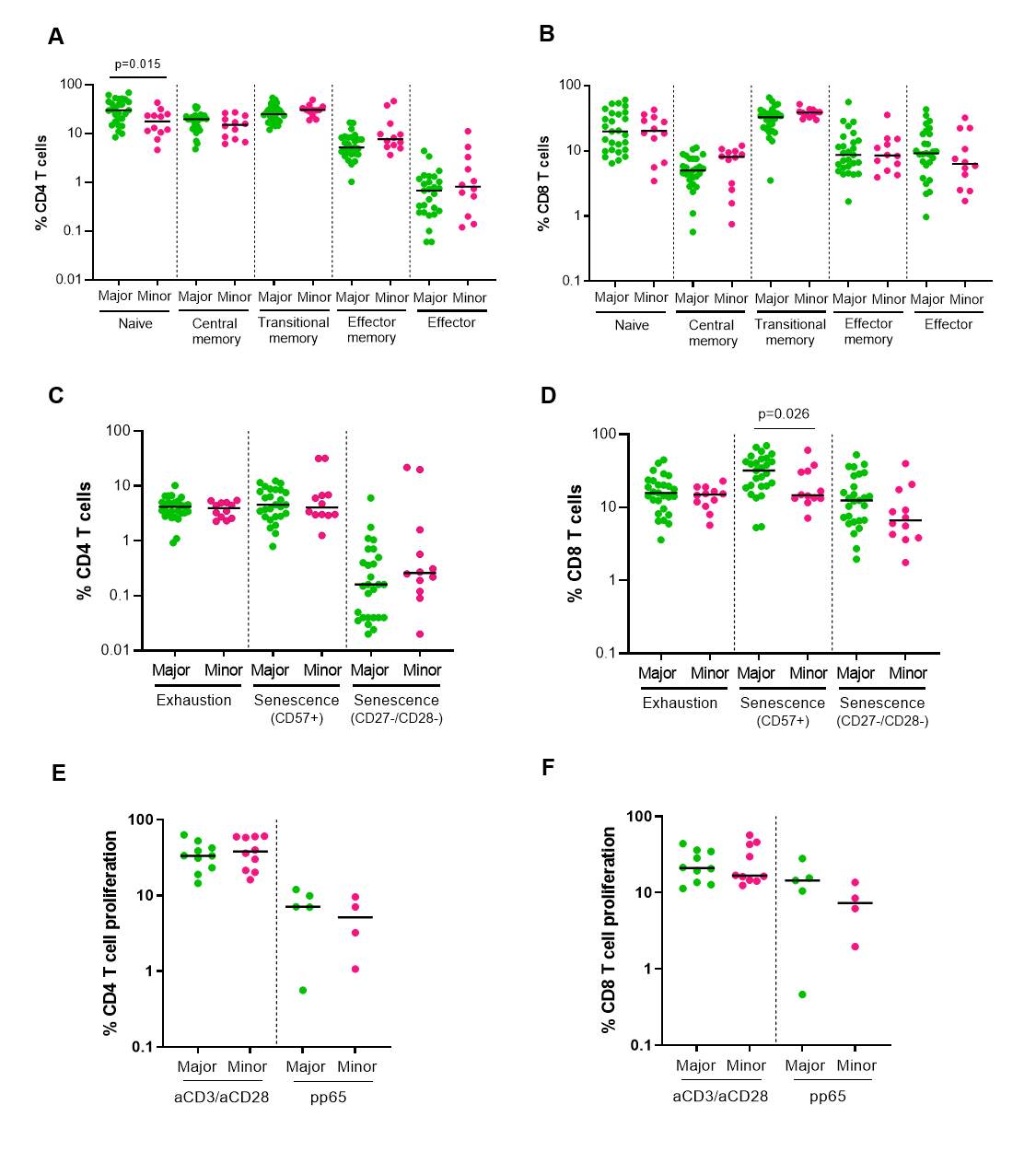


**Supplementary figure 4: The effect of SNP rs4131564 on T cell differentiation, exhaustion, senescence and proliferation in blood donors.** Flowcytometry analysis of PBMC from blood donors carrying the major or minor allele of rs4131564: CD4 and CD8 T cell differentiation as determined by CD45RA, CD27 and CCR7 expression(A&B); exhaustion as determined by PD-1 expression and senescence as determined by CD57 expression or loss of CD27 and CD28 expression (C&D)**;** Percentage CD4 and CD8 T cell proliferation as determined by the loss of CellTrace Violet, upon 6 days of culture with anti-CD3/CD28 or CMV pp65 peptide pool stimulation (E&F).

**Supplementary figure 5: The effect of SNP rs4131564 on T cell cytokine expression in PWH.** Flowcytometry analysis of intracellular cytokine production in CD4 (A-E) and CD8 (F-J) T cells of PWH carrying the major or minor allele of rs4131564 after stimulation with SEB or HIV-1 gag peptide pool.
